# Diverging Trends in Recent Population-Based Survival Rates in Oesophageal and Gastric Cancer

**DOI:** 10.1371/journal.pone.0041352

**Published:** 2012-07-18

**Authors:** Jesper Lagergren, Fredrik Mattson

**Affiliations:** 1 Upper Gastrointestinal Research, Department of Molecular Medicine and Surgery, Karolinska Institutet, Stockholm, Sweden; 2 Division of Cancer Studies, King's College London, United Kingdom; Sookmyung Women's University, Republic of Korea

## Abstract

**Background:**

Survival trends in oesophageal and gastric cancer need to be updated. A nationwide Swedish population-based study in 1961–2009 was based on registry data.

**Methodology/Principal Findings:**

Relative survival rate, i.e. the ratio of the observed to the expected survival, adjusted for age, sex, and calendar period, and presented with 95% confidence intervals (CI), was the main outcome measure. The expected survival was calculated using the corresponding Swedish general population with no exclusions. The relative survival rates in oesophageal and gastric cardia adenocarcinoma have improved since the 1990s (p for trend <0.001), but not in oesophageal squamous cell carcinoma or gastric non-cardia adenocarcinoma. The relative 5-year survival rates during the two recent periods 1990–1999 and 2000–2008 were 12.5% (95%CI 10.1%–14.9%) and 10.3% (95%CI 8.5–12.0%) for oesophageal squamous cell carcinoma, 12.5% (95%CI 10.1%–14.9%) and 14.6% (95%CI 12.6–16.6%) for oesophageal adenocarcinoma, 11.1% (95%CI 9.6%–12.6%) and 14.3% (95%CI 12.3–16.3%) for gastric cardia adenocarcinoma, and 20.2% (95%CI 19.2%–21.1%) and 19.0% (95%CI 17.7–20.2%) for gastric non-cardia adenocarcinoma. The 3-year survival in tumour stage III in 2004–2008 was about 25% for all four tumour types.

**Conclusions/Significance:**

The survival in oesophageal and cardia adenocarcinoma is increasing, but the lack of such increase in oesophageal squamous cell carcinoma and gastric non-cardia adenocarcinoma is a concern.

## Introduction

A shared characteristic of cancers of the oesophagus and the stomach is the poor overall prognosis. The reported overall 5-year survival rate in oesophageal cancer in Europe is about 10%, and the corresponding rate for gastric cancer is about 20% [1]. There is a need to assess potential changes in the prognosis of these tumours. Sweden offers excellent opportunities to assess population-based survival in cancer. There is a complete assessment of all individuals through the personal identity numbers; [2] the Swedish Cancer Register has a complete recording of oesophageal and gastric cancer; [3], [4] data on dates of death and emigration is complete by virtue of the Register of the Total Population. Therefore, survival studies from Sweden can be conducted in a population-based design. We have previously reported the survival rates in oesophageal cancer in Sweden until year 1996, showing that the relative 5-year survival had improved from about 5% to about 10% in both squamous cell carcinoma and adenocarcinoma of the oesophagus during the 1990s [5]. The present study was conducted to provide recent population-based survival rates in oesophageal and gastric cancers by histological type and by subsite.

## Materials and Methods

### Ethics statement

The study was approved by the Regional Ethical Review Board in Stockholm.

### Study design

This was a nationwide Swedish population-based cohort study of the standardised relative survival rates in oesophageal squamous cell carcinoma, oesophageal adenocarcinoma, gastric cardia adenocarcinoma, and gastric non-cardia adenocarcinoma. All residents in Sweden were eligible for the study. The total study period included the years 1961–2008, with follow-up until end of 2009. The focus of the present study was on the period after 1996, i.e. the years after those included in our previous publication addressing oesophageal cancer survival. [5] The Swedish Cancer Register was used to identify all cases of oesophageal or gastric cancer. The Swedish Register of the Total Population was used to identify dates of mortality, and to exclude person-time no longer at risk of cancer recorded in the Cancer Register due to emigration.

### Data collection

This Swedish Cancer Register was established in 1958 and has an at least 96% complete registration of the type and date of diagnosis of all cancers in Sweden since 1961 [6]. The completeness for both oesophageal and gastric cancer is 98% according to comprehensive validation studies [3], [4]. The 2% non-registration is mainly due to missing of sending in the forms for registration to the Swedish Cancer Registry. The Cancer Register used the 7^th^ version of the International Classification of Diagnoses (ICD-7) for coding of cancer diagnoses. Oesophageal cancer was coded as ICD-7 code 150, while gastric cardia cancer was coded as 151.1, and gastric non-cardia was coded as 151.0, 151.8 or 151.9. The histology code (WHO/HS/CANC/24.1 Histology Code) for adenocarcinoma was 096, while it was 146 for squamous cell carcinoma. Table 1 shows how the ICD-7 and WHO/HS/CANC/24.1 coding systems compare to the ICD-10 and the ICD-O topographic and morphological classifications. Only since 2004 tumour stage has been recorded in the Swedish Cancer Registry. The 6^th^ edition of the TNM stage from 2002 was used according to the Union Internationale Contre le Cancer [7]. The assessment of the staging was done by the clinician who reported the cancer case to the Cancer Registry. Data on the specific site gastric cardia cancer was available from 1970. The personal identity number was used to link all individual cohort members to the Register of the Total Population, a register with 100% completeness of dates of death and emigration. This register provided accurate dates of all deaths during the follow-up of the study cohort, and also included data on emigration.

**Table 1:** The 7^th^ and 10^th^ version of the International Classification of Diagnoses (ICD) for coding of oesophageal and gastric cancer diagnoses, and the histology codes for adenocarcinoma and squamous cell carcinoma in the WHO/HS/CANC/24.1 Histology.

Cancer type	ICD-7	ICD-10	WHO/HS/CANC/24	ICD-O
Oesophageal squamous cell carcinoma	150	C15	146	807
Oesophageal adenocarcinoma	150	C15	096	814
Gastric cardia adenocarcinoma	151.1	C16.0	096	814
Gastric non-cardia adenocarcinoma	151.0, 151.8, 151.9	C16.1, C16.2, C16.3, C16.4, C16.5, C16.6, C16.8, C16.9	096	814

Code and the ICD-O classification.

**Figure 1:**
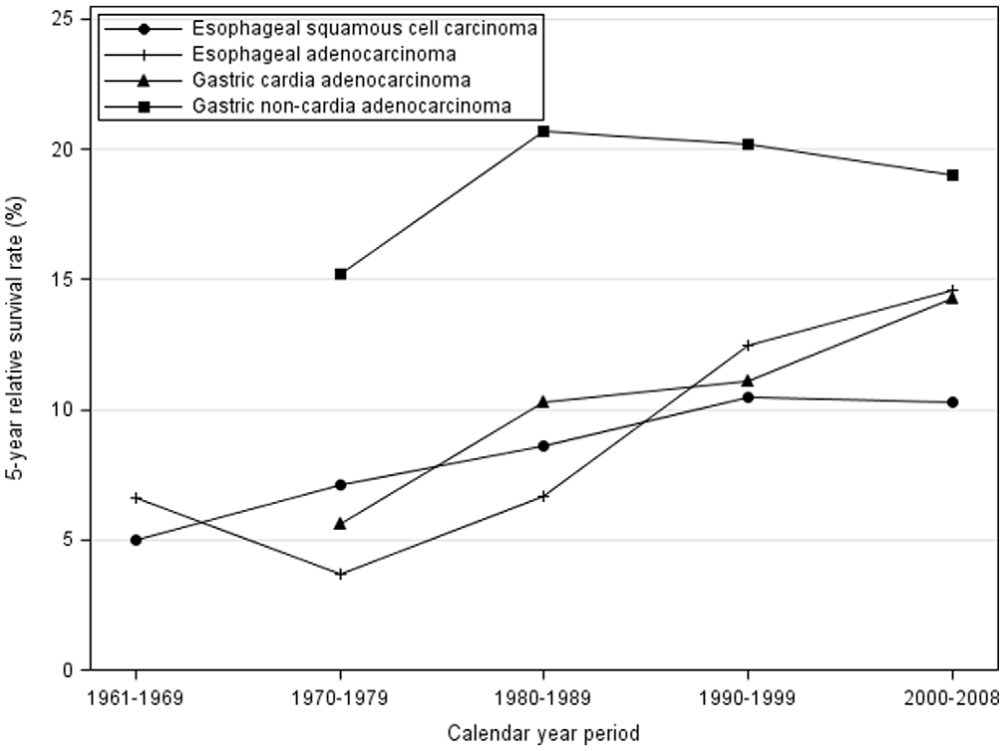
Relative survival rates (%) in defined calendar year periods of diagnosis in oesophageal squamous cell carcinoma and oesophageal adenocarcinoma in 1961–2008, and cardia adenocarcinoma and gastric non-cardia adenocarcinoma in 1970–2008, and follow-up until 2009 in Sweden.

### Statistical analysis

Observed and relative survival rates were calculated for survival 1 year, 3 years, and 5 years after diagnosis. Calendar periods were evaluated separately. For the most recent calendar period, 2000–2008, the results were also stratified for sex and age groups, while stratification for tumour stage was possible for the period 2004–2008. Observed survival rates with 95% confidence intervals (CI) were estimated using the life-table method [8]. Relative survival rates with 95% CI were the main outcome measure since it estimates the disease-specific survival. Relative survival rates were computed as the ratio of the observed to the expected survival, where the expected survival represented the survival among the entire Swedish population in the same age, sex, and calendar year. All analyses were performed using SAS Statistical Package (version 9.2, SAS Institute Inc., Gary, NC).

## Results

### Survival in oesophageal squamous cell carcinoma

Among 9,616 oesophageal squamous cell carcinomas, a majority were men (66.6%). The improvement in survival rates seen during the years 1961–1999 did not continue during the period 2000–2008 (Table 2, Figure 1). The 5-year relative survival rate was 10.5% (95% CI 9.0%–11.9%) in 1990–1999 and 10.3% (95% CI 8.5%–12.0%) in 2000–2008. The survival rates in 2000–2008 were slightly better in women compared to men, and older age was associated with worse relative survival (Table 3).

**Table 2:** Observed and relative survival rates (%) in patients with oesophageal squamous cell carcinoma and oesophageal adenocarcinoma in 1961–2008, and cardia adenocarcinoma and gastric non-cardia adenocarcinoma in 1970–2008, and follow-up until 2009 in Sweden.

				Observed survival rate (95% confidence interval)			Relative survival rate (95% confidence interval)		
Cancer	Period of diagnosis	Number of patients (%)	Mean age at diagnosis	1 year	3 years	5 years	1 year	3 years	5 years
**Esophageal squamous**	1961–1969	1414	68.9	24.8 (22.5–27.0)	6.6 (5.3–7.9)	4.3 (3.2–5.3)	26.1 (23.7–28.4)	7.5 (6.0–9.0)	5.0 (3.8–6.3)
**cell carcinoma**	1970–1979	1942	69.2	27.3 (25.4–29.3)	8.4 (7.1–9.8)	6.0 (4.8–7.1)	28.8 (26.7––30.8)	9.4 (7.9–10.9)	7.1 (5.7–8.4)
	1980–1989	2353	69.9	31.2 (29.3–33.1)	10.1 (8.7–11.5)	7.4 (6.2–8.6)	32.8 (30.8–34.7)	11.2 (9.7–12.7)	8.6 (7.2–10.0)
	1990–1999	2252	70.0	33.5 (31.6–35.5)	12.5 (11.1–14)	9.3 (8.0–10.6)	35.0 (33.0–37.1)	13.6 (12–15.2)	10.5 (9.0–11.9)
	2000–2008	1655	70.1	35.3 (33.0–37.7)	13.4 (11.6–15.1)	9.3 (7.8–10.9)	36.7 (34.3–39.1)	14.3 (12.4–16.1)	10.3 (8.5–12.0)
**Esophageal**	1961–1969	215	67.4	29.8 (23.7–35.9)	7.0 (3.6–10.4)	5.6 (2.5–8.6)	31.1 (24.7–37.5)	7.8 (4.0–11.6)	6.6 (3.0–10.2)
**adenocarcinoma**	1970–1979	360	69.3	27.2 (22.6–31.8)	6.1 (3.5–8.7)	3.2 (1.3–5.1)	28.7 (23.9–33.6)	6.8 (3.9–9.8)	3.7 (1.4–5.9)
	1980–1989	531	70.1	29.4 (25.5–33.3)	7.6 (5.1–10.1)	5.9 (3.7–8.1)	31.1 (27.0–35.2)	8.3 (5.6–11)	6.7 (4.2–9.2)
	1990–1999	909	70.3	35.6 (32.5–38.8)	16.4 (13.9–18.8)	10.6 (8.5–12.6)	37.4 (34.2–40.7)	18.1 (15.4–20.8)	12.5 (10.1–14.9)
	2000–2008	1707	69.6	40.1 (37.7–42.4)	17.1 (15.2–19.0)	13.1 (11.3–14.8)	41.8 (39.4–44.2)	18.4 (16.4–20.4)	14.6 (12.6–16.6)
**Gastric cardia**	1970–1979	951	68.3	27.3 (24.5–30.2)	8.8 (6.9–10.7)	4.9 (3.4–6.3)	28.7 (25.7–31.7)	9.7 (7.6–11.7)	5.6 (3.9–7.3)
**adenocarcinoma**	1980–1989	1563	69.9	36.6 (34.2–39.0)	12.6 (10.8–14.3)	8.8 (7.3–10.3)	38.5 (36.0–41.0)	13.8 (11.9–15.8)	10.3 (8.5–12.0)
	1990–1999	1981	69.9	37.1 (34.9–39.2)	13.8 (12.2–15.3)	9.6 (8.3–10.9)	38.9 (36.7–41.2)	15.2 (13.5–16.9)	11.1 (9.6–12.6)
	2000–2008	1743	69.2	41.0 (38.7–43.3)	17.5 (15.6–19.3)	12.4 (10.7–14.1)	42.7 (40.3–45.1)	19.1 (17.0–21.1)	14.3 (12.3–16.3)
**Gastric non–cardia**	1970–1979	15520	70.1	35.1 (34.4–35.9)	16.8 (16.2–17.5)	12.5 (11.9–13.1)	37.2 (36.4–38.0)	19.0 (18.2–19.7)	15.2 (14.5–15.9)
**adenocarcinoma**	1980–1989	14260	72.1	43.7 (42.9–44.5)	22.3 (21.5–23.1)	16.9 (16.2–17.6)	46.5 (45.6–47.3)	25.3 (24.5–26.2)	20.7 (19.9–21.6)
	1990–1999	9619	72.8	42.0 (41.0–43.0)	21.9 (21.0–22.7)	16.5 (15.7–17.3)	44.5 (43.5–45.6)	24.9 (23.9–25.8)	20.2 (19.2–21.1)
	2000–2008	5876	73.1	41.4 (40.2–42.7)	20.4 (19.3–21.5)	15.7 (14.7–16.8)	43.8 (42.5–45.1)	23.0 (21.8–24.2)	19.0 (17.7–20.2)

**Table 3:** Relative survival rates (%) in patients with oesophageal squamous cell carcinoma, oesophageal adenocarcinoma, gastric cardia adenocarcinoma and gastric non-cardia adenocarcinoma in Sweden in 2000–2008, and follow-up until 2009.

					Relative survival rate (95% confidence interval)		
Cancer	Sex and age groups			Number of patients (%)	1 year	3 years	5 years
**Esophageal squamous**	Sex	Men		1025 (61.9)	35.6 (32.6–38.6)	13.1 (10.9–15.4)	9.6 (7.5–11.7)
**cell carcinoma**		Women		630 (38.1)	38.6 (34.7–42.5)	16.1 (12.9–19.2)	11.4 (8.4–14.4)
	Age (years)	<60		301 (18.2)	50.4 (44.7–56.1)	21.0 (16.2–25.8)	16.0 (11.4–20.6)
		60–69		454 (27.4)	40.8 (36.2–45.3)	15.8 (12.2–19.4)	13.0 (9.4–16.6)
		70–79		555 (33.5)	34.1 (30.0––38.1)	12.9 (9.8–16.0)	8.9 (6.0–11.7)
		≥80		345 (20.8)	22.1 (17.4––26.9)	9.7 (5.7–13.8)	3.7 (0.1–7.6)
**Esophageal**	Sex	Men		1386 (81.2)	42.6 (39.9–45.3)	19.1 (16.9–21.4)	15.3 (13.0–17.5)
**adenocarcinoma**		Women		321 (18.8)	38.1 (32.6–43.6)	15.4 (11.1–19.8)	11.7 (7.5–15.9)
	Age (years)	<60		344 (20.2)	48.5 (43.2–53.8)	21.7 (17.1–26.2)	18.9 (14.4–23.5)
		60–69		485 (28.4)	48.4 (43.9–52.9)	24.5 (20.4–28.5)	20.8 (16.8–24.9)
		70–79		494 (28.9)	40.5 (36.0–44.9)	18.4 (14.6–22.1)	13.6 (9.8–17.3)
		≥80		384 (22.5)	27.5 (22.7–32.4)	7.8 (4.1–11.6)	3.8 (0.3–7.2)
**Gastric cardia**	Sex	Men		1331 (76.4)	44.2 (41.4–47.0)	19.5 (17.2–21.8)	14.7 (12.4–17.0)
**adenocarcinoma**		Women		412 (23.6)	37.9 (33.0–42.7)	17.7 (13.6–21.7)	13.1 (9.2–17.1)
	Age (years)	<60		384 (22.0)	51.5 (46.5–56.5)	22.4 (18.0–26.7)	17.1 (12.8–21.3)
		60–69		453 (26.0)	48.3 (43.6–52.9)	23.3 (19.2–27.5)	17.4 (13.4–21.5)
		70–79		512 (29.4)	40.4 (36.1–44.8)	19.0 (15.3–22.8)	14.2 (10.4–18.1)
		≥80		394 (22.6)	29.1 (24.2–34.0)	11.6 (7.5–15.7)	10.8 (5.5–16.1)
**Gastric non–cardia**	Sex	Men		3329 (56.7)	44.6 (42.8–46.4)	23.2 (21.6–24.9)	19.1 (17.5–20.7)
**adenocarcinoma**		Women		2547 (43.3)	42.7 (40.7–44.7)	22.6 (20.8–24.4)	18.8 (16.9–20.6)
	Age (years)	<60		826 (14.1)	52.1 (48.7–55.5)	28.6 (25.5–31.8)	23.8 (20.6–26.9)
		60–69		1137 (19.3)	48.9 (45.9–51.8)	25.6 (22.9–28.3)	19.9 (17.2–22.6)
		70–79		1897 (32.3)	44.0 (41.7–46.3)	24.7 (22.6–26.9)	21.5 (19.3–23.7)
		≥80		2016 (34.3)	36.5 (34.2–38.8)	18.1 (15.8–20.3)	15.7 (13.2–18.2)

### Survival in oesophageal adenocarcinoma

Among 3,722 patients with oesophageal adenocarcinoma, the majority (80.2%) were males. The survival rates improved during the entire study period, including the last decade (Table 2, Figure 1) (p for trend <0.001). The relative 5-year survival was 14.6% (95% CI 12.6%–16.6%) in 2000–2008, while it was 12.5% (95% CI 10.1%–14.9%) in 1990–1999. The recent survival rates (2000–2008) were higher in men compared to women, and older age groups had worse survival (Table 3).

### Gastric cardia adenocarcinoma

The cohort included 6,238 patients with gastric cardia adenocarcinoma, of whom 76.4% were men. The survival patterns were similar to those of oesophageal adenocarcinoma, with improved rates during the entire study period, including the most recent period (Table 2, Figure 1). The 5-year relative survival was 11.1% (95% CI 9.6%–12.6%) in 1990–1999 and 14.3% (95% CI 12.3%–16.3%) in 2000–2008. The recent survival rates were higher among men and younger patients (Table 3).

### Gastric non-cardia adenocarcinoma

Gastric non-cardia adenocarcinoma was found in 45,275 patients, of whom 59.5% were men. After 1980, the survival rates slightly declined (Table 2, Figure 1). The relative 5-year survival was 19.0% (95% CI 17.7%–20.2%) in 2000–2008, while it was 20.2% (95% CI 19.2%–21.1%) in 1990–1999. The recent survival rates were similar in men and women, and age did not strongly influence the relative survival (Table 3).

### Tumour stage-specific survival

Data on tumour stage during the period 2004–2008 revealed that tumour stage was strongly correlated with the relative survival in each of the four types of cancer studied (Table 4). The frequencies of missing data on tumour stage were 28.8%, 28.3%, 24.8%, and 27.3% for oesophageal squamous cell carcinoma, oesophageal adenocarcinoma, gastric cardia adenocarcinoma, and gastric non-cardia adenocarcinoma, respectively. The most advanced tumour stage (IV) represented the largest group of patients in each of the tumour groups, while a low frequency of patients (range 5.8–8.2%) were identified in the earliest tumour stage (I). The 3-year relative survival rates in tumours of stage I ranged from 68.9% for gastric non-cardia adenocarcinoma to 46.7% for oesophageal squamous cell carcinoma. The survival in patients with stage III tumours was similar, about 25%, in all tumour types (Table 4).

**Table 4:** Tumour-stage specific relative survival rates (%) in patients with oesophageal squamous cell carcinoma, oesophageal adenocarcinoma, gastric cardia adenocarcinoma and gastric non-cardia adenocarcinoma in Sweden in 2004–2008, and follow-up until 2009.

				Relative survival rate (95% confidence interval)	
Cancer	Tumour stage	Number of patients (%)	Mean age at diagnosis	1 year	3 years
**Oesophageal squamous**	Total	867	70.0	36.8 (33.5–40.1)	16.2 (13.5–19.0)
**cell carcinoma**	I	36 (4.2)	69.1	68.6 (52.8–84.5)	46.7 (27.9–65.5)
	II	188 (21.7)	69.0	48.3 (41.0–55.7)	21.6 (15.0–28.3)
	III	178 (20.5)	69.5	43.4 (36.0–50.9)	23.5 (16.8–30.2)
	IV	215 (24.8)	68.6	25.3 (19.4–31.3)	5.9 (2.3–9.6)
	Missing	250 (28.8)	72.3	28.6 (22.8–34.4)	10.8 (6.4–15.3)
**Oesophageal**	Total	1030	69.5	41.3 (38.2–44.4)	19.2 (16.5–21.8)
**adenocarcinoma**	I	52 (5.0)	67.7	79.5 (67.7–91.3)	65.7 (51.6–79.8)
	II	176 (17.1)	69.8	59.2 (51.5–66.8)	31.4 (23.3–39.4)
	III	239 (23.2)	69.1	52.4 (45.9–59.0)	26.1 (20.0–32.2)
	IV	272 (26.4)	67.3	21.3 (16.3–26.2)	3.6 (0.9–6.3)
	Missing	291 (28.3)	72.1	33.2 (27.5–38.8)	11.6 (7.4–15.7)
**Gastric cardia**	Total	960	68.9	45.7 (42.4–48.9)	21.9 (19.0–24.8)
**adenocarcinoma**	I	43 (4.5)	69.0	70.0 (55.4–84.5)	59.8 (43.5–76.2)
	II	192 (20.0)	69.0	70.8 (64.0–77.7)	46.3 (38.4–54.2)
	III	226 (23.5)	67.6	63.6 (57.0–70.1)	25.5 (19.1–31.9)
	IV	261 (27.2)	66.9	19.7 (14.8–24.6)	4.0 (0.9–7.1)
	Missing	238 (24.8)	72.1	32.5 (26.3–38.6)	10.2 (5.9–14.5)
**Gastric non-cardia**	Total	3001	73.2	44.1 (42.2–45.9)	24.3 (22.6–26.1)
**adenocarcinoma**	I	179 (6.0)	73.0	79.7 (73.2–86.3)	68.9 (60.7–77.1)
	II	509 (17.0)	73.0	69.9 (65.5–74.2)	51.2 (46.2–56.3)
	III	614 (20.5)	72.5	52.4 (48.2–56.6)	25.2 (21.3–29.0)
	IV	879 (29.3)	70.8	22.4 (19.6–25.2)	4.7 (3.1–6.4)
	Missing	820 (27.3)	76.3	37.3 (33.8–40.8)	17.4 (14.3–20.4)

## Discussion

This study indicates that the overall survival rates in oesophageal and gastric cardia adenocarcinoma have improved during the last decade, while such survival rates in oesophageal squamous cell carcinoma and gastric non-cardia adenocarcinoma have been unchanged and declined, respectively. The current (2000–2008) relative 5-year survival rates are about 10%, 15%, 15%, and 20% for oesophageal squamous cell carcinoma, oesophageal adenocarcinoma, gastric cardia adenocarcinoma, and gastric non-cardia adenocarcinoma, respectively.

Methodological strengths include the population-based design, the completeness of the assessment of tumours and mortality, as well as the calculation of relative survival which represents disease-specific mortality. Among limitations, clinical variables were not available, including treatment, co-morbidity, and other prognostic factors. The purpose of the study was, however, to evaluate only the overall survival, independent of clinical factors. Nevertheless, we did analyse the tumour stage-specific survival, which was available from 2004, although a proportion of data was missing.

The diverging patterns of survival between the studied tumours are intriguing. We can only speculate about possible reasons for these changes, particularly since we lack data on the treatment. The dramatic changes in incidence of oesophageal and gastric cancer during recent decades might for example contribute to these findings. The incidence of oesophageal and gastric cardia adenocarcinoma has increased markedly, while the incidence of gastric non-cardia adenocarcinoma and oesophageal squamous cell carcinoma has decreased over many years.[9]–[11] An increased awareness of tumours near the gastro-oesophageal junction among clinicians might have contributed to earlier detection. Moreover, the increased incidence might have increased the clinical experience in the treatment of these tumours, including surgical procedures and postoperative care. An increased centralisation of the surgical treatment of oesophageal cancer to fewer and larger centres has occurred in many countries during the recent few years, including Sweden, and this might have contributed to the better survival rates in oesophageal adenocarcinoma, but the lack of such improvement in oesophageal squamous cell carcinoma argue against this possibility.[12], [13] Another potentially relevant factor regarding long-term results in the treatment of oesophageal and gastric cancer is an increased implementation of multi-disciplinary approach and multidisciplinary team meetings, which might result in more accurate clinical decision-making and a better tailoring of the treatment for the individual patient.[14] However, the differences in survival patterns between the tumours reported in this study do not entirely fit with the influence of multi-disciplinary approach.

In agreement with the present study, a recent population-based study from 1988-2005 in the US found similar survival rates and changes in survival over time in oesophageal and cardia adenocarcinoma, which indicate that these tumours might not be distinct disease entities.[15] A small population-based study from Minnesota in the United States showed no strong changes in survival with time in oesophageal or gastric adenocarcinoma during the period 1971–2000.[16] Our previous population-based study of oesophageal cancer survival showed an improved relative survival during the 1990s compared to previous decades for both adenocarcinoma and squamous cell carcinoma.[17]

The reasons behind the changing trends in survival in these tumours should be addressed in future research. The influence of the treatment, co-morbidities and other known prognostic factors should be included in studies addressing explanations for the time trends in the overall survival.

In conclusion, this population-based study reveals an encouraging improvement in the overall relative survival rates in oesophageal and cardia adenocarcinoma, while no such improvement was found for oesophageal squamous cell carcinoma or gastric non-cardia adenocarcinoma.

## References

[1] Sant M, Allemani C, Santaquilani M, Knijn A, Marchesi F (2009). EUROCARE-4. Survival of cancer patients diagnosed in 1995–1999. Results and commentary.. Eur J Cancer.

[2] Ludvigsson JF, Otterblad-Olausson P, Pettersson BU, Ekbom A (2009). The Swedish personal identity number: possibilities and pitfalls in healthcare and medical research.. Eur J Epidemiol.

[3] Ekstrom AM, Signorello LB, Hansson LE, Bergstrom R, Lindgren A (1999). Evaluating gastric cancer misclassification: a potential explanation for the rise in cardia cancer incidence.. J Natl Cancer Inst.

[4] Lindblad M, Ye W, Lindgren A, Lagergren J (2006). Disparities in the classification of esophageal and cardia adenocarcinomas and their influence on reported incidence rates.. Ann Surg.

[5] Sundelof M, Lagergren J, Ye W (2008). Patient demographics and lifestyle factors influencing long-term survival of oesophageal cancer and gastric cardia cancer in a nationwide study in Sweden.. Eur J Cancer.

[6] Barlow L, Westergren K, Holmberg L, Talback M (2009). The completeness of the Swedish Cancer Register: a sample survey for year 1998.. Acta Oncol.

[7] Greene FL, Sobin LH (2002). The TNM system: our language for cancer care.. J Surg Oncol.

[8] Cutler SJ, Ederer F (1958). Maximum utilization of the life table method in analyzing survival.. J Chronic Dis.

[9] Lagergren J, Mattsson F (2011). No further increase in the incidence of esophageal adenocarcinoma in Sweden.. Int J Cancer.

[10] Cook MB, Chow WH, Devesa SS (2009). Oesophageal cancer incidence in the United States by race, sex, and histologic type, 1977–2005.. Br J Cancer.

[11] Brown LM, Devesa SS, Chow WH (2008). Incidence of adenocarcinoma of the esophagus among white Americans by sex, stage, and age.. J Natl Cancer Inst.

[12] Stitzenberg KB, Meropol NJ (2010). Trends in centralization of cancer surgery.. Ann Surg Oncol.

[13] Wouters MW, Karim-Kos HE, le Cessie S, Wijnhoven BP, Stassen LP (2009). Centralization of esophageal cancer surgery: does it improve clinical outcome?. Ann Surg Oncol.

[14] Davies AR, Deans DA, Penman I, Plevris JN, Fletcher J (2006). The multidisciplinary team meeting improves staging accuracy and treatment selection for gastro-esophageal cancer.. Dis Esophagus.

[15] Whitson BA, Groth SS, Li Z, Kratzke RA, Maddaus MA (2010). Survival of patients with distal esophageal and gastric cardia tumors: a population-based analysis of gastroesophageal junction carcinomas.. J Thorac Cardiovasc Surg.

[16] Crane SJ, Locke GR, 3rd, Harmsen WS, Zinsmeister AR, Romero Y, et al (2008). Survival trends in patients with gastric and esophageal adenocarcinomas: a population-based study.. Mayo Clin Proc.

[17] Sundelof M, Ye W, Dickman PW, Lagergren J (2002). Improved survival in both histologic types of oesophageal cancer in Sweden.. Int J Cancer.

