# Correction: Diverging Trends in Recent Population-Based Survival Rates in Oesophageal and Gastric Cancer

**DOI:** 10.1371/annotation/ec5f9404-d890-4963-b722-2518f9e1ec82

**Published:** 2012-08-13

**Authors:** Jesper Lagergren, Fredrik Mattsson

There was a typographical error in the second author's name. Fredrik Mattsson is correct. The correct citation is:

Lagergren J, Mattsson F (2012) Diverging Trends in Recent Population-Based Survival Rates in Oesophageal and Gastric Cancer. PLoS ONE 7(7): e41352. 

